# Adverse events in small dogs treated with a single dose of vinblastine

**DOI:** 10.1093/jvimsj/aalag050

**Published:** 2026-03-30

**Authors:** Fukiko Matsuyama, Kei Harada, Masanao Ichimata, Eri Fukazawa, Ryuzo Katayama, Tetsuya Kobayashi

**Affiliations:** Japan Small Animal Cancer Center, Japan Small Animal Medical Center, 1-10-4, Higashitokorozawa wada, Tokorozawa-shi, Saitama 359-0023, Japan; Japan Small Animal Cancer Center, Japan Small Animal Medical Center, 1-10-4, Higashitokorozawa wada, Tokorozawa-shi, Saitama 359-0023, Japan; Japan Small Animal Cancer Center, Japan Small Animal Medical Center, 1-10-4, Higashitokorozawa wada, Tokorozawa-shi, Saitama 359-0023, Japan; Japan Small Animal Cancer Center, Japan Small Animal Medical Center, 1-10-4, Higashitokorozawa wada, Tokorozawa-shi, Saitama 359-0023, Japan; Japan Small Animal Cancer Center, Japan Small Animal Medical Center, 1-10-4, Higashitokorozawa wada, Tokorozawa-shi, Saitama 359-0023, Japan; Japan Small Animal Cancer Center, Japan Small Animal Medical Center, 1-10-4, Higashitokorozawa wada, Tokorozawa-shi, Saitama 359-0023, Japan

**Keywords:** adverse events, vinblastine, small dogs, dose intensity, febrile neutropenia

## Abstract

**Background:**

Vinblastine (VBL) is a chemotherapeutic agent commonly used to treat malignant tumors in dogs. Although a maximum tolerated dose of 3.5 mg/m^2^ has been reported, the safety and tolerability of this dose in small dogs remain inadequately characterized.

**Hypothesis/Objectives:**

Retrospectively evaluate adverse events (AEs) associated with a single IV administration of VBL at a dosage of 2.0 mg/m^2^ in dogs weighing < 10 kg. We hypothesized that AE occurrences in small dogs would be comparable to those reported in phase I studies involving larger breeds.

**Animals:**

Forty client-owned dogs weighing < 10 kg with malignant tumors.

**Methods:**

Adverse events after a single IV VBL administration at a dosage of 2.0 mg/m^2^ were retrospectively evaluated in small dogs with malignant tumors. Myelosuppressive and gastrointestinal AEs were graded using VCOG-CTCAE v2.0 criteria.

**Results:**

Neutropenia and thrombocytopenia were observed in 82.5% (33/40) and 5.0% (2/40) of dogs, respectively. Grade 3 or 4 neutropenia occurred in 32.5% (13/40), comprising 15.0% (6/40) with Grade 3 and 17.5% (7/40) with Grade 4 events. Inappetence, vomiting, and diarrhea were reported in 7.5% (3/40), 7.5% (3/40), and 25.0% (10/40) of dogs, respectively, and were generally mild. Febrile neutropenia was noted in 5.0% (2/40) of dogs.

**Conclusions and clinical importance:**

Vinblastine administered at a dosage of 2.0 mg/m^2^ in small dogs with malignant tumors resulted in gastrointestinal toxicity within clinically acceptable limits, but close monitoring for neutropenia is warranted. These findings suggest that dose escalation of VBL, as reported in larger dogs, may not be feasible in smaller dogs.

## Introduction

Vinblastine (VBL) is a vinca alkaloid that binds to tubulin in microtubules, thereby disrupting spindle formation and inducing cell cycle arrest.^1–3^ It is commonly used in dogs for the treatment of various malignant tumors.^4–10^ Historically, it has been employed in the management of mast cell tumors,^4–8^^,^^11^ but more recently, its efficacy has also been reported in urothelial carcinoma^9^^,^^12^ and large cell lymphoma.^10^^,^^13^^,^^14^ Several studies have empirically used VBL at a dosage of 2.0 mg/m^2^, typically administered every 1-2 weeks.^4^^,^^5^

Because of the relatively low incidence of adverse events (AEs) observed at this dosage, a phase I dose-escalation study was conducted in 2008, increasing the dosage to 3.0, 3.5, and 4.0 mg/m^2^ q2 weeks.^14^ The maximum tolerated dose (MTD) was determined to be 3.5 mg/m^2^ q2 weeks.^14^ However, the median body weight of dogs enrolled in that study was 35.5 kg, and none weighed <10 kg. Conversely, a study evaluating VBL in dogs with transitional cell carcinoma (TCC) found a higher incidence of neutropenia in dogs weighing <15 kg treated at 3.0 mg/m^2^ (75%), compared with those ≥15 kg (23.1%).^9^ These findings suggest that small breed dogs may have increased susceptibility to VBL-related toxicity and that alternative dosing strategies may be warranted.

In veterinary oncology, chemotherapeutic doses typically are calculated based on body surface area (BSA) rather than body weight to account for inter-breed size variation. However, several studies have shown that small dogs may experience a higher incidence of AEs despite BSA-based dosing.^15–20^ Agents such as doxorubicin, mitoxantrone, melphalan, and carboplatin are known to require lower recommended doses in small breed dogs than in larger breeds.^17–20^ Although the pharmacologic profile of VBL has been well described in large dogs,^21^ its safety in smaller dogs has not been adequately investigated.

Our objective was to retrospectively evaluate AEs after a single administration of VBL at 2.0 mg/m^2^ in tumor-bearing dogs weighing <10 kg. We hypothesized that the frequency of AEs in these small dogs would be comparable to that reported in phase I studies involving larger dogs.

## Materials and methods

### Study animals

A retrospective review was conducted of dogs weighing <10.0 kg presented to the Japan Small Animal Cancer Center, affiliated with the Japan Small Animal Medical Center Foundation, between May 2012 and December 2022. Dogs were eligible for inclusion if they had a cytologic or histopathologic diagnosis of malignancy and received VBL at a dosage of 2.0 mg/m^2^.

Dogs were excluded if they had a prior history of VBL administration, were receiving concurrent cytotoxic chemotherapy or molecularly targeted therapy, or exhibited severe clinical signs attributable to gastrointestinal disease or the primary tumor, such as mast cell tumors with Darier’s signs or systemic illness. Dogs also were excluded if they were suspected of having clinically relevant hepatic failure, had a pre-treatment total bilirubin concentration exceeding 1.5 times the upper limit of the reference interval,^21^ or belonged to breeds with a high likelihood of the MDR1 gene mutation (ie, Collies and Australian Shepherds). In addition, dogs that had received toceranib or imatinib within 4 days before VBL treatment were excluded. However, dogs that had received prior cytotoxic chemotherapy were included if the interval between their most recent treatment and VBL administration exceeded the standard dosing interval.

### Study design

A dose of VBL equivalent 2.0 mg/m^2^ was added to 5-10 mL of normal saline and administered IV over approximately 5 min. Data collected included signalment, tumor type, concomitant medications, presence or absence of febrile neutropenia (FN), and AEs related to myelosuppression and gastrointestinal toxicity.

Myelosuppression was assessed by evaluating the severity and frequency of neutropenia and thrombocytopenia. Febrile neutropenia was defined as a body temperature ≥39.2 °C with a neutrophil count ≤2500 cells/μL.^16^ Gastrointestinal toxicity was evaluated by scoring appetite on a 10-point scale, with owners documenting the daily frequency of vomiting and diarrhea.

We evaluated the frequency of hematologic and gastrointestinal toxicities according to the presence or absence of macroscopic lesions.

The observation period was defined as the interval between VBL administration and the next chemotherapy treatment. All toxicities were graded according to the Veterinary Cooperative Oncology Group–Common Terminology Criteria for Adverse Events (VCOG-CTCAE v2.0).^22^

### Statistical analysis

The frequency of grade ≥3 neutropenia (severe neutropenia) was compared between dogs weighing below and above the median, as well as between those younger and older than the median age,^23^ using a χ^2^ test. Statistical significance was defined as *P* < .05. Analyses were performed using Stata Statistical Software, version 14.2 (StataCorp, College Station, TX, United States).

## Results

### Study demographics

Forty dogs were included in the study. All dogs that received VBL were included in the evaluation of AEs. The median age was 9.5 years (range, 5-15 years) and median body weight was 4.8 kg (range, 1.7-9.4 kg). The study population consisted of 8 intact males, 9 neutered males, 4 intact females, and 19 spayed females. Breeds represented included Chihuahua (*n* = 6), Jack Russell Terrier (*n* = 5), Toy Poodle (*n* = 5), Miniature Dachshund (*n* = 5), mixed breed (*n* = 3), Maltese (*n* = 3), Miniature Schnauzer (*n* = 2), Pug (*n* = 2), Shiba Inu (*n* = 2), and one each of English Bulldog, Boston Terrier, French Bulldog, Italian Greyhound, Shih Tzu, Pomeranian, and Yorkshire Terrier. Tumor types included cutaneous or SC mast cell tumors (*n* = 34), TCC of the bladder (*n* = 4), nasal mast cell tumor (*n* = 1), and gastrointestinal mast cell tumor involving the small intestine, ileum, and liver (*n* = 1). At the time of VBL administration, 18 dogs had macroscopic disease, whereas 22 had microscopic disease only.

Concomitant medications included prednisolone (*n* = 25), enrofloxacin (*n* = 25), maropitant (*n* = 17), famotidine (*n* = 14), diphenhydramine (*n* = 14), omeprazole (*n* = 3), cephalexin (*n* = 2), and ursodeoxycholic acid (*n* = 2). The following medications each were administered to 1 dog: ondansetron, firocoxib, lansoprazole, metronidazole, piroxicam, telmisartan, torasemide, and benazepril (some dogs received multiple agents; see Table S1). All antimicrobial agents were prescribed prophylactically at the discretion of the attending veterinarian, and maropitant likewise was administered prophylactically in all cases. With respect to the timing of administration, one dog received IV maropitant only on the day of VBL administration; 6 dogs received IV maropitant on the day of VBL treatment followed by PO administration (duration, 2 days; *n* = 1; 6 days, *n* = 5); and 10 dogs received PO maropitant only after VBL administration (duration, 4 days; *n* = 2; 5 days, *n* = 3; 6 days, *n* = 4; 14 days, *n* = 1). Given the potential for proton pump inhibitors (PPIs) to influence VBL metabolism,^21^^,^^24^ only 3 dogs received concurrent PPI treatment. In 1 dog with a cutaneous mast cell tumor presenting as a macroscopic lesion, a PPI was administered as supportive care, but no objective response to VBL was observed, and treatment was discontinued after a single dose. Another dog had a mast cell tumor without macroscopic lesions, and omeprazole was prescribed prophylactically to prevent prednisolone-associated gastrointestinal toxicosis. This dog received 8 VBL treatments without evidence of recurrence during the treatment course. In the remaining dog, a PPI had already been prescribed before VBL administration for management of firocoxib-associated gastrointestinal toxicosis. Although gastrointestinal signs had nearly resolved by the time of VBL administration, no therapeutic response to VBL was observed, resulting in discontinuation after a single dose.

Chemotherapeutic agents administered within 1 month before VBL included toceranib (*n* = 3), imatinib (*n* = 2), and bleomycin and intralesional cisplatin for electrochemotherapy (*n* = 1).

### Adverse events

**Figure 1:**
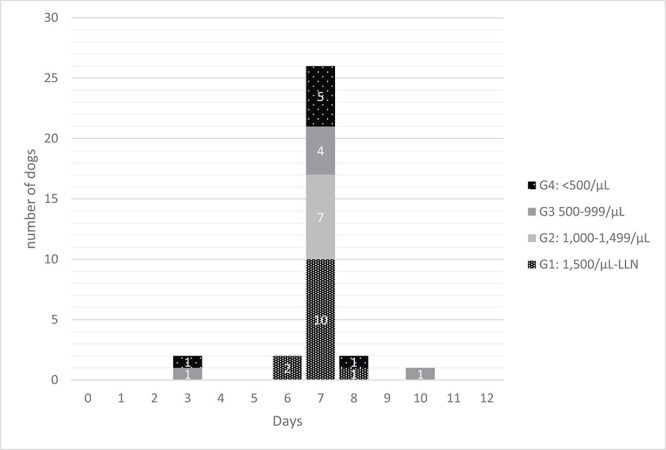
Days on which neutrophil nadirs were confirmed after vinblastine administration.

Neutropenia and thrombocytopenia were observed in 82.5% (33/40) and 5.0% (2/40) of dogs, respectively. Grade ≥ 3 neutropenia occurred in 32.5% (13/40) of dogs. The distribution of neutropenia grades is presented in Table 1. The mean and median time to neutrophil nadir were 6.9 and 7 days, respectively (range, 3-13 days). Of the 2 dogs in which neutropenia was identified on day 3, one was presented with gastrointestinal signs, whereas the other was presented for evaluation of fever and found to be neutropenic on complete blood count (CBC) performed at that visit. The day on which the neutrophil nadir was observed was day 7 in 80% (32/40) of dogs, earlier than day 7 in 10% (4/40), and later than day 7 in 10% (4/40). The distribution of nadir confirmation days is shown in Figure 1. With a minimum neutrophil count of 2000/μL required for VBL administration,^25^^,^^26^ 21 of 36 dogs (58.3%) had neutrophil counts ≤2000/μL on day 7, precluding subsequent VBL administration. Thrombocytopenia was observed in 2 dogs, both on day 7 after VBL administration. Febrile neutropenia was documented in 2 dogs (5.0%), both of which developed Grade 4 neutropenia. One dog had a mast cell tumor, and the other had TCC. Their body weights were 2.3 and 4.1 kg, respectively. The frequency of Grade ≥ 3 neutropenia was 8 of 20 dogs (40%) in dogs with body weights below the median, and 5 of 20 dogs (25%) in those with body weights at or above the median. No significant difference was observed in the frequency between the two groups (*P* = .3; 95% confidence interval [CI], 0.43-9.82).

**Table 1:** Frequency of cytopenias in small dogs (<10 kg) treated with a single dose of vinblastine (VBL) 2.0 mg/m^2^.

	VBL 2.0 mg/m^2^ (*n* = 40)	%
**Neutropenia** **Grade 0** **Grade 1** **Grade 2** **Grade 3** **Grade 4**	33 7 13 7 6 7	82.5 17.5 32.5 17.5 15 17.5
**Thrombocytopenia** **Grade 0** **Grade 1** **Grade 2** **Grade 3** **Grade 4**	2 38 1 0 1 0	5.0 95 2.5 0 2.5 0
**Febrile Neutropenia**	2	5.0

Regarding age, the frequency of Grade ≥ 3 neutropenia was 7 of 20 dogs (35%) in dogs younger than the median age, and 6 of 20 dogs (30%) in those at or above the median age. No significant difference was observed between the groups (*P* = .7; 95% CI, 0.27-5.85).

Inappetence was observed in 3 dogs. Two dogs were classified as Grade 3 and occurred concurrently with FN. The 1 dog with Grade 1 diarrhea had pre-existing soft feces before VBL administration. One dog developed Grade 3 diarrhea during FN. The distribution of gastrointestinal AEs by grade is presented in Table 2. One dog with a mast cell tumor originating in the small intestine developed Grade 2 neutropenia, but no gastrointestinal toxicities were observed.

**Table 2:** Frequency of clinical signs associated with gastrointestinal toxicosis in small dogs (<10 kg) treated with a single dose of vinblastine (VBL) 2.0 mg/m^2^.

	VBL 2.0 mg/m^2^ (*n* = 40)	%
**Anorexia** **Grade 0** **Grade 1** **Grade 2** **Grade 3** **Grade 4**	3 37 1 0 2 0	7.5 92.5 2.5 0 5 0
**Vomiting** **Grade 0** **Grade 1** **Grade 2** **Grade 3** **Grade 4**	3 37 3 0 0 0	7.5 92.5 7.5 0 0 0
**Diarrhea** **Grade 0** **Grade 1** **Grade 2** **Grade 3** **Grade 4**	10 30 6 3 1 0	25 75 15 7.5 2.5 0

The frequency of hematologic and gastrointestinal toxicities according to the presence or absence of macroscopic lesions is shown in Table 3.

**Table 3:** Frequency of adverse events according to the presence or absence of macroscopic lesions.

Gross lesions	Neutropenia	Thrombocytopenia	Anorexia	Vomiting	Diarrhea
**Yes *n* = 18 (%)**	13 (72.2)	2 (11.1)	1 (5.5)	1 (5.5)	4 (22.2)
**No *n* = 22 (%)**	20 (90.9)	0	2 (9.0)	2 (9.0)	6 (27.2)

## Discussion

Our objective was to evaluate AEs associated with a single administration of VBL at 2.0 mg/m^2^ in dogs weighing <10 kg. The findings indicate that neutropenia was the primary AE, whereas gastrointestinal toxicity generally was mild. In this cohort, the frequency of Grade ≥ 3 neutropenia was 32.5%, which is comparable to the 25% frequency reported in medium- to large-breed dogs treated with VBL at 3.5 mg/m^2^ q2 weeks.^14^ Additionally, approximately half of the dogs failed to demonstrate adequate neutrophil recovery (>2000/μL) by day 7 after VBL administration. These results suggest that weekly administration of VBL at 2.0 mg/m^2^ may not be feasible in some small breed dogs.

When chemotherapy agents are dosed based on BSA, small breed dogs may be more susceptible to AEs.^15–20^ In particular, some chemotherapy agents, such as doxorubicin, mitoxantrone, melphalan, and carboplatin, are dosed based on body weight to mitigate toxicity in small dogs.^17–20^ A previous study of VBL in dogs with TCC reported that those weighing <15 kg required dose reductions from 3.0 mg/m^2^ q2 weeks because of myelotoxicity.^9^ In our study, 32.5% of dogs developed Grade ≥ 3 neutropenia at a dosage of 2.0 mg/m^2^, further suggesting that increasing the dose above this threshold may not be tolerable for dogs weighing <10 kg.

The appropriate dosing interval for VBL also may differ based on body size and drug tolerance. In protocols for treating mast cell tumors in dogs, VBL at a dosage of 2.0 mg/m^2^ often is administered weekly in combination with prednisolone.^4^^,^^5^ However, the phase I study that established the MTD of 3.5 mg/m^2^ q2 weeks recommended a biweekly dosing interval.^14^ In our study, CBCs obtained 7 days after treatment indicated that 45% (18/40) of dogs had neutrophil counts <1500 cells/μL, precluding administration of the next dose. These findings suggest that the severity of myelosuppression at 2.0 mg/m^2^ in small dogs may approximate that seen in larger dogs treated at the MTD. At present, we consider a dosage of 2.0 mg/m^2^ every 2 weeks appropriate for small breed dogs.

Neutropenia is the primary dose-limiting toxicity of VBL,^14^^,^^27^^,^^28^ and the development of FN poses a serious clinical concern. In previous studies, FN occurred in 1 of 16 dogs (6.3%) and 2 of 26 dogs (7.7%) treated with 3.5 mg/m^2^ VBL q2 weeks.^14^^,^^29^ In our study, FN occurred in 2 of 40 dogs (5.0 %), both of which had Grade 4 neutropenia. Although the frequency is similar to prior reports, most dogs in our study received prophylactic antibiotics, which may have decreased the risk of infection. Therefore, the incidence of FN in the absence of prophylactic treatment may be underestimated.

We examined whether hematologic toxicity associated with VBL is more prevalent in dogs with lower body weights by comparing the frequency of severe neutropenia between dogs with body weights below and above the median. Although no significant difference was observed between the two groups, these findings should be interpreted with caution. The limited sample size and low number of severe neutropenia events raise the possibility of Type II error (false-negative results). Notably, both cases of FN occurred in dogs with body weights below the median. A VBL dosage lower than 2.0 mg/m^2^ may be appropriate in individual dogs, and ongoing monitoring for hematologic toxicity is warranted in small dogs receiving more than a single dose of VBL.

Vinblastine generally is regarded as well tolerated, with low rates of gastrointestinal toxicity.^14^^,^^28^^,^^29^ In a phase I study of VBL at 3.0-4.0 mg/m^2^ q2 weeks (*n* = 23), vomiting occurred in 2 dogs (Grades 1 and 2) and diarrhea in 1 dog (Grade 1).^14^ Another study using 2.0-3.0 mg/m^2^ (*n* = 24) q1 week reported vomiting in 3 dogs (Grades 1-3) and diarrhea in 3 dogs (Grades 1-2).^30^ In our study, most gastrointestinal AEs were < Grade 2 and clinically manageable. However, because prophylactic antiemetics and antidiarrheals were administered concurrently in many cases, the true incidence of gastrointestinal toxicity may have been underestimated. Future studies in dogs not receiving prophylactic medications are warranted to better assess VBL-associated gastrointestinal effects.

Our study had some limitations. First, its retrospective design and the evaluation of AEs limited to the initial administration of VBL may have introduced bias. The inclusion of cases that had received prior cytotoxic or molecularly targeted treatments could have influenced the assessment of toxicity. In addition, heterogeneity in tumor types, lack of standardized tumor staging and mast cell tumor grading, and the absence of controls on concomitant medications further limit the generalizability of our findings. The latter, in particular, may have introduced confounding variables affecting the frequency of gastrointestinal toxicity or FN. Gastrointestinal toxicity was assessed based on owner-reported observations, raising the possibility of underreporting. Moreover, because CBCs were not monitored on a daily basis after VBL administration, the exact nadirs of neutrophil and platelet counts could not be determined, which may have led to an underestimation of the actual incidence of neutropenia and thrombocytopenia.

In conclusion, AEs observed after administration of VBL at 2.0 mg/m^2^ in dogs weighing <10 kg appear comparable to those reported in larger dogs treated at the MTD of 3.5 mg/m^2^ q2 weeks. Our findings suggest that small dogs may require distinct dosing strategies. Prospective evaluation using a Phase I dose escalation study is warranted to establish an appropriate and safe dosing regimen for this population.

## Supplementary Material

Supplementary_Table_1_aalag050

## Data Availability

The data that support the findings of this study are available from the corresponding author upon reasonable request.

## Off-label antimicrobial declaration

Some dogs in this study received cefalexin and metronidazole as part of their treatment to prevent adverse events. In Japan, cefalexin and metronidazole have been approved for use in humans and animals, and both products were used in this study. The other drugs are not licensed in Japan for use in dogs. For this study, we chose to use these drugs in the dogs because they are considered safe, and we deemed them necessary for treatment.

## Institutional animal care and use committee or other approval declaration

Approved by the Institutional Ethics Committee of Japan Small Animal Medical Center (approved August 16, 2025).

## Abbreviations

AEs: adverse events
BSA: body surface area
CTZ: chemoreceptor trigger zone
FN: febrile neutropenia
GI: gastrointestinal
MTD: maximum tolerated dose
TCC: transitional cell carcinoma
VBL: vinblastine
VCOG-CTCAE v2.0: Veterinary Cooperative Oncology Group-common terminology criteria for adverse events
