# Supplementary material for: Adverse events in small dogs treated with a single dose of vinblastine
Source: J Vet Intern Med. 2026 Mar 30;40(2):aalag050. doi: 10.1093/jvimsj/aalag050 (PMC13034547; doi:10.1093/jvimsj/aalag050)
Supplement: Supplementary_Table_1_aalag050 [file supplementary_table_1_aalag050.docx]

Supplementary Table 1. Concomitant medications given to dogs in this study.
